# Bundling of cellulose microfibrils in native and polyethylene glycol-containing wood cell walls revealed by small-angle neutron scattering

**DOI:** 10.1038/s41598-020-77755-y

**Published:** 2020-11-30

**Authors:** Paavo A. Penttilä, Michael Altgen, Muhammad Awais, Monika Österberg, Lauri Rautkari, Ralf Schweins

**Affiliations:** 1 Department of Bioproducts and Biosystems, Aalto University, P.O. Box 16300, 00076 Aalto, Finland; 2 Large-Scale Structures Group, Institut Laue-Langevin (ILL), 71 Avenue des Martyrs, 38042 Grenoble, France

**Keywords:** Structure determination, Biopolymers, Characterization and analytical techniques, Polymers

## Abstract

Wood and other plant-based resources provide abundant, renewable raw materials for a variety of applications. Nevertheless, their utilization would greatly benefit from more efficient and accurate methods to characterize the detailed nanoscale architecture of plant cell walls. Non-invasive techniques such as neutron and X-ray scattering hold a promise for elucidating the hierarchical cell wall structure and any changes in its morphology, but their use is hindered by challenges in interpreting the experimental data. We used small-angle neutron scattering in combination with contrast variation by poly(ethylene glycol) (PEG) to identify the scattering contribution from cellulose microfibril bundles in native wood cell walls. Using this method, mean diameters for the microfibril bundles from 12 to 19 nm were determined, without the necessity of cutting, drying or freezing the cell wall. The packing distance of the individual microfibrils inside the bundles can be obtained from the same data. This finding opens up possibilities for further utilization of small-angle scattering in characterizing the plant cell wall nanostructure and its response to chemical, physical and biological modifications or even in situ treatments. Moreover, our results give new insights into the interaction between PEG and the wood nanostructure, which may be helpful for preservation of archaeological woods.

## Introduction

The major part of woody plants consists of secondary cell walls, which dictate their mechanical properties and accommodate most of the polysaccharides, including the main component cellulose^1^. In order to promote the utilization of wood and plant biomass in applications ranging from building materials to advanced nanomaterials^2,3^ and platform chemicals^4^, a more complete picture of the secondary cell wall structure in woody plants is needed.

In the secondary cell walls of wood, cellulose molecules form partially crystalline cellulose microfibrils, which have a lateral thickness of 2 to 3 nm^5^. These microfibrils are arranged into aggregates or bundles within a matrix formed of less ordered polysaccharides, i.e. hemicelluloses, and lignin. The exact morphology and the locations of the different components relative to each other are still not fully clear^6–8^. Water in wood occupies a considerable proportion (approximately 30% in the water-saturated state^1^) of the cell wall volume, hydrating the less ordered polysaccharides and filling the nanoscale pores between and around the microfibrils^9–11^.

Lateral aggregation of microfibrils in the form of bundles, also termed macrofibrils, in plant cell walls has been observed by various methods^12^, including scanning electron microscopy^13,14^, transmission electron microscopy^15^, atomic force microscopy^16,17^, and electron tomography^18^. Typically, lateral mean diameters around or below 20 nm have been reported for microfibril bundles in unprocessed wood cell walls^11,13,16,17^, with variation between 10 and 60 nm depending on the individual bundle, the type of wood and cell wall, and the method of determination. However, none of the methods used so far has been able to observe these structures in their truly native state, hydrated by liquid water and as part of an intact cell wall structure.

Among the methods for structural characterization of plant cell wall nanostructure^19^, small-angle neutron and X-ray scattering scattering (SANS, SAXS) are exceptional in the sense that they require only minimal sample preparation and they can be used under different environments such as temperature and relative humidity. However, the most serious challenge hampering the wider application of these methods on plant-based materials is the complicated way the different structural components contribute to the scattering intensities. Despite of being able to distinguish the scattering of regularly packed cellulose microfibrils and a power-law contribution from larger pores^20,21^, the models for small-angle scattering analysis^22^ have not been able to identify a contribution specific to higher-order aggregation of the cellulose microfibrils.

Poly(ethylene glycol) (PEG) has been used for a long time to stabilize both green and archaeological woods against moisture changes and drying^23,24^. These applications take advantage of the intrinsic tendency of PEG to be absorbed from an aqueous solution into the wood cell wall. However, the exact way how PEG stabilizes the wood structure at the nanoscale is not fully understood^23^. By impregnating wood cell walls with PEG and utilizing contrast variation in SANS, we were able to assign a specific contribution in the SANS intensities of native wood to the outer dimensions of cellulose microfibril bundles. We show that SANS can be conveniently and efficiently used to characterize the bundling of cellulose microfibrils in different wood species and to determine the bundle diameter without any physical or chemical interference in the native, hydrated cell wall structure. This is different to any of the current methods, which require cutting of the cell wall in order to expose its inner structure and allow only a small volume of the sample to be observed at once. The method provides new insights into the structural aspects of plant biomass, offering a new characterization tool for plant biology and aiding the development of new applications from wood and other plant-based biomass. At the same time, our data might help to explain the stabilization mechanism of PEG in wood cell walls.

## Results

### PEG penetrates the cell walls

In the impregnation of wood by PEG, the water-soluble PEG molecules penetrate the mesopores of the wood cell wall, replacing water in the pores and thus keeping the structure fixed upon the removal of water^1^. Certain limiting size for PEG to be effective in this process has been suggested, even though the proposed values vary considerably for instance based on the exact procedure of the impregnation and its evaluation^25^. Due to the chemical similarity of PEG and the cell wall constituents, observing the interactions between PEG and the wood cell wall nanostructure suffers from similar challenges as studies of the native cell walls. In such situations, highly useful information can be obtained by spatially-resolved spectroscopic methods such as Raman imaging combined with multivariate image analysis^26^. Moreover, combining spectroscopic imaging with scattering methods offers a powerful set of tools for resolving the penetration depth of PEG in the hierarchical structure of wood.

**Figure 1:**
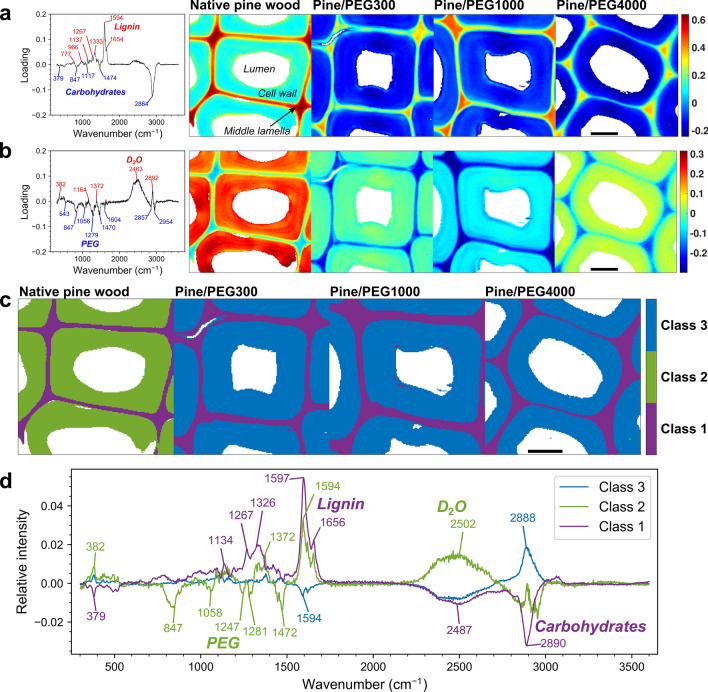
Confocal Raman spectroscopy results from pine wood in ${\text {D}}_2\text {O}$ with and without PEG (scale bars 10 ${\upmu }$m). (**a**) Loading plot (left) and score image (right) of principal component 1, which differentiates mainly between regions rich in lignin (positive) and carbohydrates (negative). (**b**) Loading plot (left) and score image (right) of principal component 2, differentiating mainly between regions rich in ${\text {D}}_2\text {O}$ (positive) and PEG (negative). Loading plots and score images of the other principal components are shown in Supplementary Fig. S1a,b,c. (**c**) Cluster image based on principal component analysis with 5 components and 3 classes (number of each chosen based on Supplementary Fig. S1d,e). The cell wall regions in the PEG-containing samples belong to class 3, whereas that of the native wood belongs to class 2. (**d**) Average mean-centered Raman spectra corresponding to each class. Bands related to PEG (Supplementary Fig. S1f) particularly show negative contribution in class 2 (native wood cell walls), meaning that PEG was present in the cell walls of all samples except the native wood. The positive contribution of ${\text {D}}_2\text {O}$ bands (around $2500~{\hbox {cm}}^{-1}$) in the same class (class 2) indicates that PEG replaced some of the ${\text {D}}_2\text {O}$ in the cell walls.

We impregnated the cell walls of wood samples representing three common Northern wood species, birch, spruce and pine, with PEG of different average degrees of polymerization. This was done by immersing the wood samples in ${\text {D}}_2\text {O}$ (for reasons detailed later) and gradually increasing the concentration of PEG in the solution up to 60%. The size of the PEG molecules, with average molar masses ranging from 300 to 4,000 g/mol (denoted PEG300, PEG1000 and PEG4000), was originally thought to influence their penetration into the cell wall. In order to investigate and confirm the presence of PEG in the wood cell walls, we conducted confocal Raman spectroscopy imaging and multivariate image analysis on latewood cells of pine wood with and without PEG (Fig. 1 and Fig. S1 in the Supplementary Information). The overlapping Raman bands of wood and PEG were separated by principal component analysis of an image mosaic, where the first five principal components explained 86% of the variation within the dataset. The analysis clearly showed that, given sufficient time, all of the PEGs could impregnate the cell walls, irrespective of their molecular weight.

The first principal component (Fig. 1a) showed mainly differences between the lignin-rich middle lamella and the carbohydrate-rich cell walls. In addition, within the cell wall regions of the PEG-containing samples, the contribution of PEG to the CH/${\text {CH}}_2$ stretching bands at 2700 to $3020~{\hbox {cm}}^{-1}$ (see Supplementary Fig. S1f for spectra from PEG in ${\text {D}}_2\text {O}$) decreased the scores of the first principal component as compared to the cell walls of the native wood. The second principal component (Fig. 1b) showed opposite contributions for bands related to ${\text {D}}_2\text {O}$ and PEG (Supplementary Fig. S1f), which confirms that PEG was present in the cell wall regions of all the PEG-containing samples and that the PEG molecules replaced ${\text {D}}_2\text {O}$ in the cell walls of these samples. The remaining three principal components did not provide further information on the PEG within the wood cell wall. Instead, they were related to differences in cellulose microfibril angle (band around $1091~{\hbox {cm}}^{-1}$)^27^ across the cell wall (third and fourth principal component, Supplementary Fig. S1a,b) and residual pixels with high contribution from the PEG solution (fifth principal component, Supplementary Fig. S1c).

The scores of the first five principal components were also utilized in a cluster analysis, which highlighted the categorical difference between the control sample and the PEG-containing ones (Fig. 1c,d). In particular, the cell wall regions of wood in pure ${\text {D}}_2\text {O}$ belonged to a different class (class 2) than those of the PEG-containing samples (class 3), whereas the lignin-rich middle lamella belonged to the same class (class 1) in all samples. Therefore, it is evident based on the Raman spectroscopy results that PEGs with molecular weights up to 4,000 g/mol could penetrate the cell walls rather evenly, and when doing so, they at least partly replaced water in the pores of the cell walls. However, the Raman imaging data alone does not show down to which level of the hierarchical cell wall structure the PEGs penetrated. This is a task that neutron scattering with contrast variation can solve.

### SANS reveals the microfibril bundles

**Figure 2:**
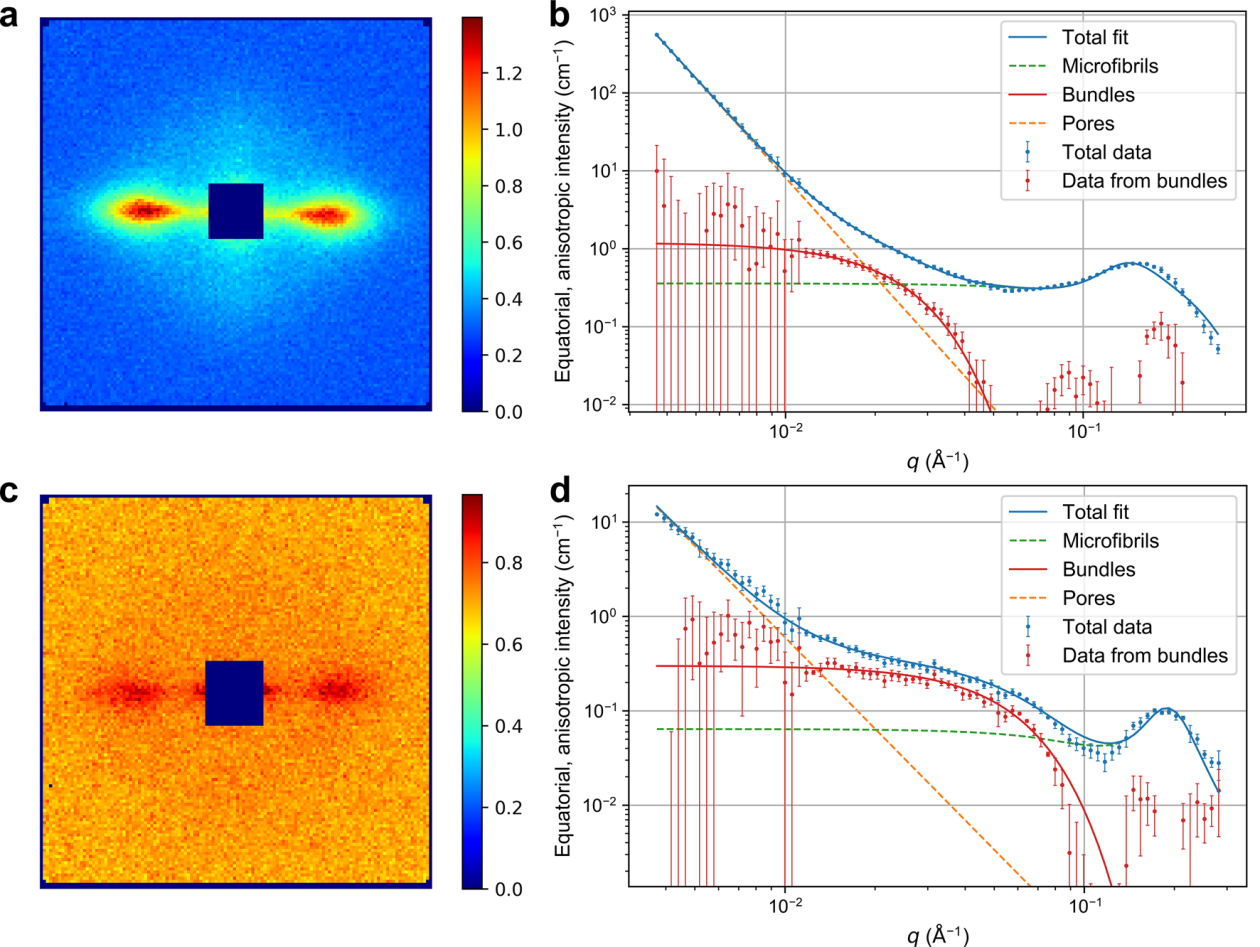
SANS data from wood with and without PEG. (**a,b**) Pine wood in pure ${\text {D}}_2\text {O}$. (**c,d**) Pine wood in a solution of 60% PEG1000 in ${\text {D}}_2\text {O}$. The proposed contribution from the microfibril bundles and those previously assigned to individual cellulose microfibrils and larger water-filled pores and cell lumina^22^ are shown in (**b,d**) (fitting parameters in Supplementary Table S3). Two-dimensional SANS patterns corresponding to the *q* range from 0.06 to $0.3~\AA ^{-1}$ are presented in (**a,c**). The equatorial, anisotropic intensity corresponds to the roughly horizontal intensity profile in the two-dimensional patterns, from which the isotropic component at each value of *q* has been subtracted.

When illuminated by a neutron or X-ray beam, a wood sample exhibits a strongly anisotropic scattering pattern (Fig. 2a) corresponding to structures in the nanometer scale. The equatorial scattering intensity profile, which appears in the horizonthal plane perpendicular to the wood fiber axis, exhibits scattering from the lateral cross-section of well-oriented fibrillar structures. Particularly in wet wood, the equatorial SANS and SAXS intensities carry information on the lateral dimensions and packing of individual cellulose microfibrils. A model to interpret this data and to extract parameters describing such features was recently presented^22^, and it has been used to follow in situ the effects of moisture changes on the microfibrils and their packing with SAXS^28^. Nevertheless, the model includes a previously unassigned contribution, which appears in a similar way in normal softwoods and hardwoods, and which we have been now able to link to the average lateral size of the microfibril bundles.

As a special possibility of SANS, the sensitivity of neutrons to different isotopes allows the technique of contrast variation^29^. Usually, this refers to eliminating or enhancing the scattering of one or more components in a multicomponent system, by adjusting its scattering length density via modifying its isotopic composition. In the case of wood samples, this technique is often used to enhance the contrast between crystalline microfibrils and water by replacing ${\text {H}}_2\text {O}$ by ${\text {D}}_2\text {O}$^5,30^. However, in the current case, our idea was to eliminate the scattering from larger water-filled pores by filling them with a water-soluble polymer having a scattering length density comparable to that of crystalline cellulose and lignin (Supplementary Table S1). The size of water-filled pores in the wood cell wall^10,31^ is similar to the size of PEG molecules in ${\text {D}}_2\text {O}$ solution, which we determined to vary between 0.5 and 2 nm at concentrations from 5 to 20 wt% (Supplementary Table S2 and Fig. S2). Thus, impregnating the wood cell walls with PEG enabled us to enhance the scattering from smaller ${\text {D}}_2\text {O}$-filled pores inside and immediately around the microfibril bundles, which were too small to allow the access of the PEG molecules.

Figure 2 presents a comparison of SANS data between samples in pure ${\text {D}}_2\text {O}$ (Fig. 2a,b) and in 60% solution of PEG1000 in ${\text {D}}_2\text {O}$ (Fig. 2c,d). Figure 2b,d show the equatorial, aniostropic scattering intensities of the same samples, which have been obtained from the two-dimensional patterns (Fig. 2a,c) by integrating azimuthally around the equatorial streaks and subtracting the isotropic scattering contribution at each value of the scattering vector *q*. Additionally, contributions previously assigned to the microfibrils and larger pores^22^ have been subtracted from the experimental data in order to highlight the remaining contribution, which dominates the intensity in the *q* range $0.01{-}0.05~\AA ^{-1}$. This contribution (denoted “Data from bundles” in Fig. 2b,d) appeared similar in both types of samples, regardless of the wood species and the presence or absence of PEG (Supplementary Fig. S3).

In order to approximate the contribution denoted “Data from Bundles” in Fig. 2b,d by a simple mathematical model, it was fitted with a Gaussian function having its maximum at $q = 0~\AA ^{-1}$. The Gaussian function was further considered equal to the Guinier law^32^, which describes the dimensions of particles or particle-like structures that are spatially separated from each other. Even though this approximation may not be strictly valid in the case of wood cell wall structure with aggregated microfibril bundles, it was considered sufficient to describe the bundle diameter in the absence of any clear indications of a correlation peak in the SANS data. By taking into account the assumed cylindrical shape of the scattering structures, the width of the Gaussian function (Supplementary Table S3) could be related to structural features with a lateral diameter of 12–19 nm in the native woods in pure ${\text {D}}_2\text {O}$ and 8–9 nm in samples containing PEG. The similar shape of the contribution in both types of samples indicates that it arises from the same structural elements, which we propose to correspond to microfibril bundles described previously in the literature^12^.

As small-angle scattering is sensitive only to differences in scattering length density between phases, it is necessary to ensure that the scattering contribution proposed to arise from the microfibril bundles does not correspond to aligned pores instead. In support of our interpretation, however, the relative intensity of the contribution here assigned to the microfibril bundles ($q = 0.01{-}0.1~\AA ^{-1}$) in comparison to the peak from the packing of the microfibrils ($q = 0.1{-}0.2~\AA ^{-1}$) is almost not at all affected by the PEG impregnation. Based on the size of the PEG molecules (Supplementary Table S2) and the Raman spectroscopy results (Fig. 1), it is assumed that any pores of at least a few nanometres in diameter would be filled by PEG. Due to the similar scattering length density of PEG and the matrix polymers in ${\text {D}}_2\text {O}$ (Supplementary Table S1), these PEG-filled pores should not contribute significantly to the SANS intensity. In particular, it is unlikely that such pores would contribute to the scattering in a similar way under two very different contrast situations, i.e. with and without PEG. In other words, if the contribution assigned here to microfibril bundles would instead originate from pores, we would expect filling of the pores with PEG to decrease the contrast and diminish the intensity contribution. Therefore, given its presence in all the wood species and PEG conditions studied here, we conclude that the referred contribution in the equatorial SANS data from wood samples originates from the regular size of microfibril aggregates in the cell wall.

### PEG influences the packing of microfibrils

**Figure 3:**
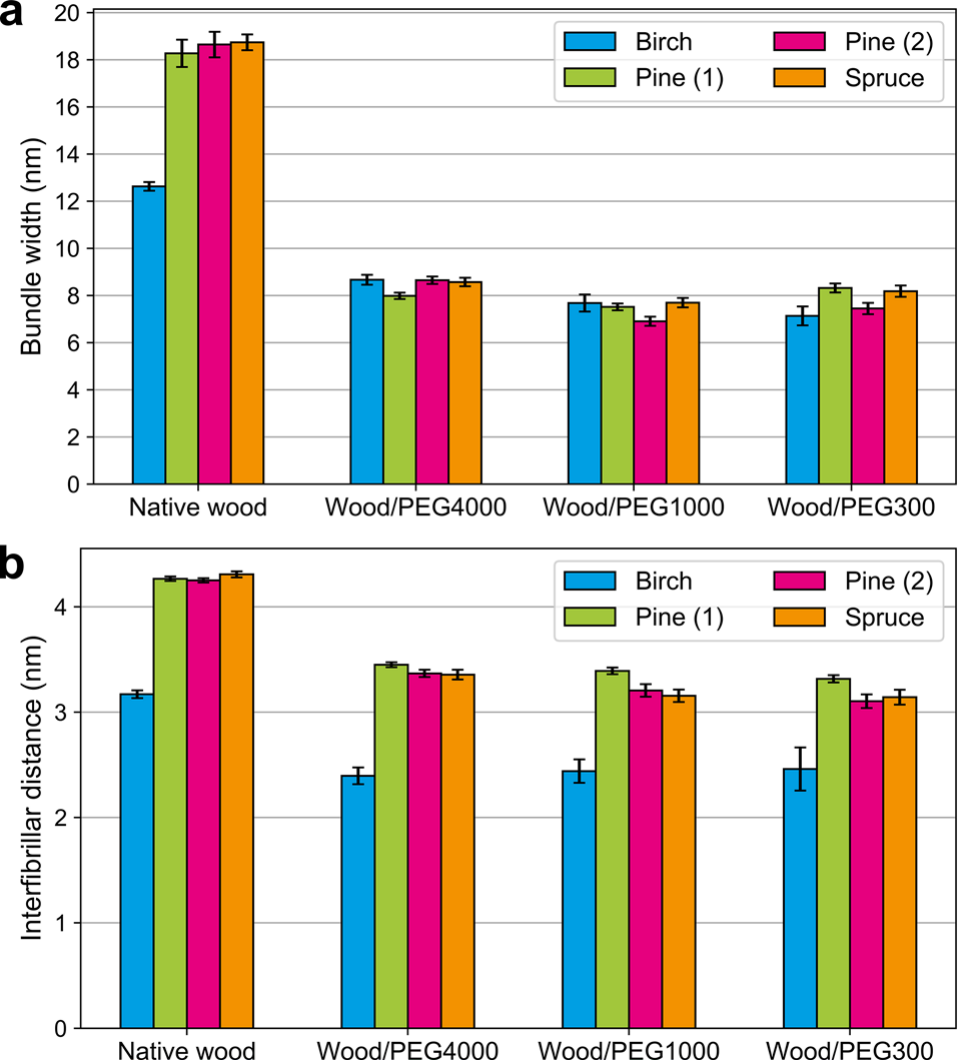
SANS results from wood samples with and without PEG. (**a**) Diameter of microfibril bundles. (**b**) Lateral center-to-center spacing between individual cellulose microfibrils. All fitting parameters according to the model of reference^22^ are presented in Supplementary Table S3.

Although the general shape and relative strength of the scattering intensity feature associated to the microfibril bundles remained largely unchanged regardless of the presence or absence of PEG, it shifted to higher *q* values by the inclusion of PEG (Supplementary Fig. S4). This shift was reflected in the calculated values of the microfibril bundle diameter (Fig. 3a), which showed a clear decrease in all samples as a consequence of PEG impregnation. At the same time, the peak attributed to the distance between cross-sectional centre points of neighboring cellulose microfibrils showed a similar shift (Supplementary Fig. S4), interpreted as a shorter interfibrillar distance and denser packing of the microfibrils in the presence of PEG (Fig. 3b). A decrease in the microfibril packing distance is typically associated with drying of especially softwoods^22,30,33^, in which case the peak decreases in intensity due to the removal of contrasting water from between the microfibrils. In our PEG-containing samples, however, the peak appeared even more clearly after the introduction of PEG, which indicates that water remained inside of the microfibril bundles and between the individual microfibrils. In addition, SANS data from wood samples impregnated with PEG4000 but immersed in fresh ${\text {D}}_2\text {O}$ (Supplementary Fig. S4 and Table S3) showed partially swollen microfibril bundles, which is explained by some of the PEG escaping from the cell walls to the surrounding ${\text {D}}_2\text {O}$ solution. This finding consolidates our interpretation that the bundle width and interfibrillar distance, both determined from SANS data, are interrelated and depend on the presence of PEG in the cell wall.

**Figure 4:**
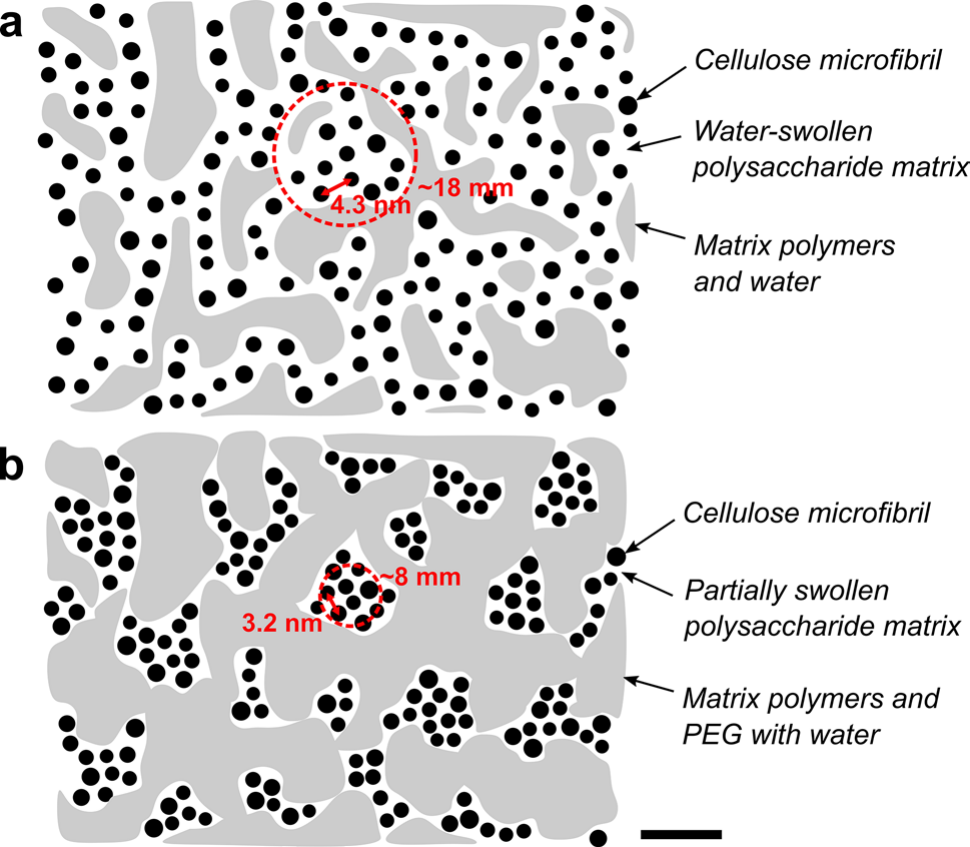
Sketches of the cross-sectional secondary cell wall structure of softwood (based on reference^18^), with the average circumference of a microfibril bundle indicated by a circle and the distance between microfibrils by an arrow (scale bar 10 nm). (**a**) Water-swollen, native state. (**b**) Wood impregnated with PEG.

The observed changes caused by the presence of PEG evoke speculations that the PEG molecules would not stabilize the structure against drying deformations merely by occupying the water-filled pores they are able to penetrate. Instead, PEG molecules also seem to attract water from inside of the microfibril bundles, which causes the bundles to de-swell. This was observed as a radical decrease of both the interfibrillar distance and the bundle diameter especially in the softwood samples (Fig. 3). A sketch to illustrate these changes is presented in Fig. 4. The differences caused by PEG were less dramatic but still significant in birch wood, which was the only representative of hardwoods in this study. This might be related to the overall smaller wet-state interfibrillar distance reported for hardwoods based on SANS and SAXS data^22,28,34^. In general, the results support previous findings from pulp fibers^17^, according to which the PEG molecules mostly occupy the spaces between microfibril bundles, but are not able to penetrate into the polymeric matrix between the individual microfibrils.

## Discussion

By using PEG to reduce the scattering from water-filled pores outside of the cellulose microfibril bundles in wood, we were able to assign a specific contribution in the SANS data from wood samples to the regular diameter of microfibril bundles. Extracting the bundle width from SANS data is relatively straightforward, and, perhaps most importantly, it can be determined for samples in their native, water-saturated state and without any need to modify the cell wall structure.

Based on our results from the native, never-dried softwood samples, the cellulose microfibrils with a centre-to-centre distance of about 4.3 nm are aggregated into bundles having an average diameter of 18–19 nm. In excellent agreement with our results, mean values of 18 nm and 19 nm have been previously determined with atomic force microscopy^11,16^ and scanning electron microscopy^13^, respectively, for the thickness of microfibril bundles in unprocessed softwoods. In the birch sample, on the other hand, the microfibrils were only 3.2 nm apart from each other and formed bundles with an average diameter of 13 nm. This difference is in line with other studies that have reported smaller interfibrillar distances^22,28,34^ and bundle diameters^13,14^ in hardwoods. The origin of these differences is not yet clear^14^, but they probably come down to the details of the biosynthesis and lignification of the secondary cell wall. One possible explanation is the different hemicellulose composition between these two types of wood, which may affect the formation of supramolecular assemblies of cellulose and their interaction with water^35^.

In addition to the SANS data presented in this paper, the current interpretation of the Gaussian-shaped scattering contribution can be extended to previous results analysed using the same model^22,28^. Such analyses of water-saturated softwood samples including spruce^22,28^, pine^22^ and fir^28^ yield bundle widths mostly between 17 and 19 nm, whereas those of hardwoods birch^22^ and beech^28^ are lower at 11–14 nm. These values are similar to those obtained for native, water-saturated woods in this study, including the smaller bundle width in hardwoods as compared to softwoods. On the other hand, drying decreased the bundle widths in spruce and pine to around 8 nm^22^, which is identical to the bundle width in the current PEG-containing samples. This might reflect a maximum packing density of microfibrils in the bundles, limited by physical constraints. Regarding the stabilizing function of PEG in the wood cell wall, this information could give new insights into cultural heritage conservation and other similar applications. At the same time, the sensitivity of the bundle width to the moisture content in wood might at least partly explain the large variation of the bundle widths in the literature^12^.

Finally, it should be noted that in the case of tension wood, the interpretation of a scattering contribution around the *q* range of the bundles may be different. This is due to a stronger contribution of nanoscale pores^28,36^ that dominates the scattering at *q* values $0.01{-}0.1~\AA ^{-1}$. Also, when fitting SAXS data from wood, the interpretation of the Gaussian contribution^22^ still requires full validation due to the different conditions of scattering length density contrast for X-rays as compared to neutrons. So far, SAXS intensity contributions resembling those originating from microfibril bundles have been detected in delignified woods only^37,38^. This implies that the possibility to observe microfibril bundles in native wood via small-angle scattering is exclusively reserved to the use of neutrons, which provide favorable scattering length density constrasts between the different wood components.

Based on the results of this work, SANS can be used to simultaneously determine the average packing distance of microfibrils and the diameter of microfibril bundles from wet wood samples without the necessity of cutting or drying the cell walls. This possibility opens up interesting opportunities for future studies. These could include in situ experiments to follow the effects of varied moisture conditions, temperature, mechanical loads, as well as more complicated procedures like physico-chemical pretreatments of biomass even at high temperatures and pressures. Understanding the effects of such procedures on the nanoscale structure of biomass is topical for the current development of sustainable, zero-waste biorefineries. For the field of plant biology, this technique offers an easy and non-destructive way to study the native bundling of microfibrils at least in different wood species if not across a wider spectrum of plants or genetically engineered phenotypes. Furthermore, it could shed light on complex phenomena related to cell wall biosynthesis, such as the formation of microfibrils and microfibril bundles, and secondary wall lignification.

## Methods

### Wood samples and PEG impregnation

Samples from *Betula pubescens* (birch), *Pinus sylvestris* (samples from two individual trees, denoted pine (1) and pine (2)) and *Picea abies* (spruce) were collected from the outer part of the xylem of mature, living trees in Eastern Finland and stored refrigerated in 30% ethanol solution^22^. Approximately 1 mm (tangential) $\times \, 10 \, \hbox {mm}$ (radial) $\times \, 10{-}15 \, \hbox {mm}$ (longitudinal) pieces of the wood samples were cut with a razor blade and immersed in 15 ml ${\text {D}}_2\text {O}$. The samples were stored at room temperature for 1 day, after which the ${\text {D}}_2\text {O}$ was exchanged, and the samples were stored at $7^\circ \hbox {C}$ for 6 days. The PEG treatment was started by immersing the wood pieces from ${\text {D}}_2\text {O}$ to about 18 ml of 10% (w/v) PEG in ${\text {D}}_2\text {O}$ in 40-ml glass vials. PEGs of three different molecular weights, BioUltra 300 (PEG300), BioUltra 1000 (PEG1000) and BioUltra 4,000 (PEG4000), were all obtained from Sigma-Aldrich, Inc. The glass vials were placed in a roller mixer at room temperature and the PEG concentration was increased up to 60% with about 1-week intervals (except for a 3-week stay at 30%). The air remaining in the glass vials was replaced by ${\text {N}}_2$ gas prior to closing. After reaching the target concentration of 60% (w/v), the samples were left in the roller mixer for 6 to 8 months prior to the SANS experiments. The samples were stored at room temperature for another 3 months prior to the Raman imaging, except for the sample with PEG4000, which was stored for 19 months.

### SANS measurements and data analysis

SANS experiments^39–41^ were carried out at the D11 instrument of the Institut Laue-Langevin in Grenoble, France. Wood sections with the cell axis aligned vertically were immersed in quartz glass cuvettes with a light path of 2 mm. Each cuvette was filled with the PEG solution from the corresponding sample. The wood samples in PEG4000 solution were additionally measured after immersing them in fresh ${\text {D}}_2\text {O}$ about 3 hours prior to data collection. Separate measurements of PEG solutions in ${\text {D}}_2\text {O}$ were done using similar cuvettes with a lightpath of 1 mm. Up to three detector positions, with sample-to-detector distances of 1.4 or 1.5 m, 8 m, and 34 or 39 m, and wavelength $\lambda = 6.0~{\AA }$ were used to cover a total range of $q= 0.002{-}0.3~\AA ^{-1}$ for the magnitude of the scattering vector $q = 4 \pi \sin {\theta }/\lambda$ (with scattering angle $2\theta$). Normalization of the two-dimensional scattering patterns to absolute units was done using the Large Array Manipulation Program (LAMP). Separation of equatorial, anisotropic scattering and azimuthal integration in the case of wood samples was done as described previously^22^, followed by merging and rebinning using Python scripts. Fitting to the equatorial, anisotropic intensities was done using the SasView software^42^ together with the freely-available WoodSAS plugin^22^, and the bundle width was obtained as described in the Supplementary Information. Data from the pure PEG solutions was treated in LAMP and fitted using Python scripts.

### Confocal Raman imaging and analysis

Transversal sections with a thickness of 20 ${\upmu }$m were cut from the pine (2) samples (with and without PEG) using a rotary microtome. The sections were placed on glass slides together with a droplet of PEG solution from the corresponding sample, covered with a glass coverslip (thickness 0.17 mm), and sealed with nail polish. Raman mapping was performed using a confocal Raman microscope (WITec alpha 300 RA, WITec, Germany) equipped with a 532-nm frequency-doubled Nd:YAG laser and a DU970-BV EMCCD camera behind a 600 lines/mm grating. Raman images^43^ with a size of $45~{\upmu }\hbox {m} \, \times \, 45~{\upmu }\hbox {m}$ and 175 lines per image and 175 points per line were acquired with a $100\times$ immersion-oil objective (numerical aperture 1.25, coverslip correction 0.17 mm). The integration time was set to 0.3 s. For the multivariate image analysis, Raman images from different samples were combined into an image mosaic. The image mosaic was unfolded into a two-dimensional data matrix with individual pixels and corresponding wavenumbers as row objects and columns, respectively. Wavenumbers outside the region $300{-}3600~{\hbox {cm}}^{-1}$ were excluded. Cosmic ray removal, baseline correction and vector normalization were performed as described previously^26^. Principal component analysis was done after mean-centering of the spectra and the first principal component was used to exclude pixels in the cell lumen based on a score value threshold. The remaining data set was mean-centered and the principal component analysis was recalculated. The first five principal components were also used in a scores-based clustering approach for an effective grouping of the pixels. The partitional K-means clustering segregated the pixels based on their correlation with the mean of each cluster. A predefined number of clusters was chosen based on the mean correlation of pixels with the respective cluster centroids (Supplementary Fig. S1e) and Euclidean distance was used to select the first centroids furthest away from the centre of the score space. The scores and pixel classes were refolded back to the image dimension to allow visualization of the results. Multivariate data analysis was performed through a combination of in-house Matlab (MathWorks, Inc.) scripts and commercial functions from the PLS Toolbox (Eigenvector Research, Inc.).

## Supplementary information

Supplementary Information.

## Data Availability

The data that support the findings of this study are available from the corresponding author upon reasonable request. The SANS data is available in the data portal of Institut Laue-Langevin (ILL), 10.5291/ILL-DATA.INTER-378. The confocal Raman mapping data is available in the Zenodo repository, 10.5281/zenodo.4094665.
