# Supplementary material for: Bundling of cellulose microfibrils in native and polyethylene glycol-containing wood cell walls revealed by small-angle neutron scattering
Source: Sci Rep. 2020 Nov 30;10:20844. doi: 10.1038/s41598-020-77755-y (PMC7705696; doi:10.1038/s41598-020-77755-y)
Supplement: Supplementary file 1 — Supplementary Information. [file 41598_2020_77755_MOESM1_ESM.pdf]

Bundling of cellulose microfibrils in native and polyethylene glycol-containing wood cell walls revealed by small-angle neutron scattering

Supplementary information

Paavo A. Penttilä[1,2,*], Michael Altgen[1], Muhammad Awais[1], Monika Österberg[1], Lauri Rautkari[1], Ralf Schweins[2]

[1]Aalto University, Department of Bioproducts and Biosystems, P.O. Box 16300, FI-00076 Aalto, Finland
[2]Institut Laue–Langevin (ILL), Large-Scale Structures Group, 71 Avenue des Martyrs, F-38042 Grenoble, France
* Corresponding author (paavo.penttila@aalto.fi)

Model for fitting SANS data

The model used to fit the equatorial, anisotropic SANS intensities is based on WoodSAS, a small-angle scattering data analysis model tailored for wood samples[1]:

$$I(q) = AI_{cyl}(q, \bar{R}, \Delta R, a, \Delta a) + Be^{-q^2/(2\sigma^2)} + Cq^{-\alpha} \quad \text{(S1)}$$

In equation (S1), $I_{cyl}(q)$ is the intensity from infinite cylinders in a hexagonal array with paracrystalline distortion[2], truncated at low q values[1]. $\bar{R}$ denotes the mean cylinder radius with standard deviation ΔR, and a is the distance between the cylinders' center points with paracrystalline distortion Δa. The cylinders correspond to individual cellulose microfibrils[1] and the third term of the equation, a power-law at low q with exponent $\alpha \approx 4$, to larger pores and the cell lumina[3,4]. The second term, a Gaussian function centered at $q = 0$ Å^{-1}, was assigned here to the cross-section of microfibril bundles.

Scattering from a dilute system of spherical particles with radius of gyration R_g can be approximated by the Guinier law[5]

$$I(q) = I_0 e^{(-q^2 R_g^2/3)} \quad \text{(S2)}$$

when $qR_g \ll 1$. The Guinier law for long cylindrical particles[6] is obtained from equation (S2) by replacing R_g with the cross-sectional radius of gyration $R_{g,c} = \sqrt{2/3}R_g$. By utilizing the similarity between the second term of equation (S1) and the Guinier law (equation (S2)), the width (diameter) of microfibril bundles can be calculated from σ by

$$Bundle\ width = 2\sqrt{2}R_{g,c} = 4R_g/\sqrt{3} = 2\sqrt{2}/\sigma. \quad \text{(S3)}$$

Table S1 Calculated scattering length densities (SLD) for different components present in the samples. The density values for lignin and hemicelluloses are based on molecular dynamics simulations and the moisture content refers to D_2O, according to reference 7.

Component	Chemical composition	Mass density (g/cm^3)	Neutron SLD ($\times 10^{10}$ cm^{-2})
Crystalline cellulose (microfibril core)	$C_6H_{10}O_5$	1.60	1.87
60% PEG in D_2O	57 wt% $C_2H_4O_1$/43 wt% D_2O	1.12	3.12
Lignin with MC 20%	83 wt% $C_{31}H_{34}O_{11}$/17 wt% D_2O	1.39	3.04
Hemicellulose with MC 50%	67 wt% $C_6H_{10}O_5$/33 wt% D_2O	1.29	3.46
Deuterated water	D_2O	1.11	6.39

Table S2 Results from fits of Guinier law (equation (S2)) to the SANS intensities from PEGs in D_2O (Fig. S2).

Sample	Concentration (wt.%)	I_0 (cm^{-1})	R_g (nm)
PEG300	57	0.6291(3)	0.055(2)
	20	0.2240(3)	0.225(1)
	10	0.1339(3)	0.329(3)
	5	0.0757(3)	0.385(7)
PEG1000	57	0.6589(3)	0.084(1)
	20	0.2969(4)	0.374(2)
	10	0.2221(5)	0.629(5)
	5	0.1588(5)	0.774(8)
PEG4000	$<$ 57 (non-precipitated fraction)	0.6817(6)	0.097(2)
	20	0.3538(4)	0.526(2)
	10	0.3653(7)	0.920(6)
	5	0.328(1)	1.34(1)

Table S3 Results from fits of the WoodSAS model (equation (S1)) to equatorial, anisotropic SANS intensities, with fixed parameters except $\Delta R/\bar{R} = 0.2$ indicated by an asterisk. The microfibril bundle widths shown in Fig. 3a of the main article were calculated from the values of σ according to equation (S3).

Sample	A (cm^{-1})	$2\bar{R}$ (nm)	a (nm)	$\Delta a/\bar{a}$	B (cm^{-1})	σ ($\times 10^{-2}$ Å^{-2})	C ($\times 10^{-8} cm^{-1}$)	α
**Birch**								
Native wood	2.8(1)	2.19(3)	3.17(4)	0.44(1)	5.0(1)	2.24(3)	7.0(7)	4.29(2)
Wood/PEG4000 in fresh D_2O	0.43(1)	2.09(2)	3.75(4)	0.419(7)	0.69(3)	1.80(4)	3.2(6)	3.91(4)
Wood/PEG4000	0.11(1)	2.0*	2.40(8)	0.31(2)	0.22(1)	3.26(8)	0.0*	2.91(3)
Wood/PEG1000	0.14(2)	2.0*	2.4(1)	0.30(2)	0.14(1)	3.7(2)	58(9)	3.17(3)
Wood/PEG300	0.09(2)	2.0*	2.5(2)	0.34(3)	0.126(9)	3.40(2)	48(7)	3.20(3)
**Pine (1)**								
Native wood	1.12(2)	1.89(2)	4.27(2)	0.291(3)	1.2(1)	1.55(5)	2.9(4)	4.23(3)
Wood/PEG4000 in fresh D_2O	0.374(7)	2.0*	3.66(4)	0.253(4)	0.24(1)	3.09(9)	110(30)	3.03(5)
Wood/PEG4000	0.220(5)	2.0*	3.45(2)	0.226(4)	0.219(8)	3.54(6)	67(2)	2.89(5)
Wood/PEG1000	0.280(8)	2.0*	3.39(3)	0.222(6)	0.30(1)	3.76(7)	22(7)	3.22(7)
Wood/PEG300	0.263(7)	2.0*	3.32(4)	0.234(6)	0.27(1)	3.40(8)	30(10)	3.13(7)
**Pine (2)**								
Native wood	1.08(2)	1.84(2)	4.25(2)	0.289(3)	1.5(1)	1.52(4)	3.0(4)	4.35(2)
Wood/PEG4000 in fresh D_2O	0.41(1)	1.92(2)	3.93(3)	0.304(4)	0.52(4)	1.87(5)	29(5)	3.47(3)
Wood/PEG4000	0.191(4)	2.0*	3.37(3)	0.247(6)	0.27(1)	3.27(6)	0.0*	3.00(3)
Wood/PEG1000	0.189(9)	2.0*	3.21(6)	0.238(9)	0.21(1)	4.1(1)	35(9)	3.21(5)
Wood/PEG300	0.166(9)	2.0*	3.10(6)	0.24(1)	0.18(1)	3.8(1)	33(8)	3.26(5)
**Spruce**								
Native wood	1.04(2)	2.01(2)	4.31(3)	0.348(3)	2.4(1)	1.51(3)	1.6(2)	4.40(3)
Wood/PEG4000 in fresh D_2O	0.36(1)	1.76(3)	3.74(3)	0.293(5)	0.38(1)	2.61(6)	1.7(4)	3.85(4)
Wood/PEG4000	0.120(4)	2.0*	3.36(5)	0.247(7)	0.200(9)	3.30(7)	0.0*	3.00(3)
Wood/PEG1000	0.172(8)	2.0*	3.16(6)	0.241(9)	0.206(9)	3.67(9)	3(2)	3.49(9)
Wood/PEG300	0.147(7)	2.0*	3.14(7)	0.25(1)	0.178(9)	3.5(1)	4(2)	3.4(0.1)

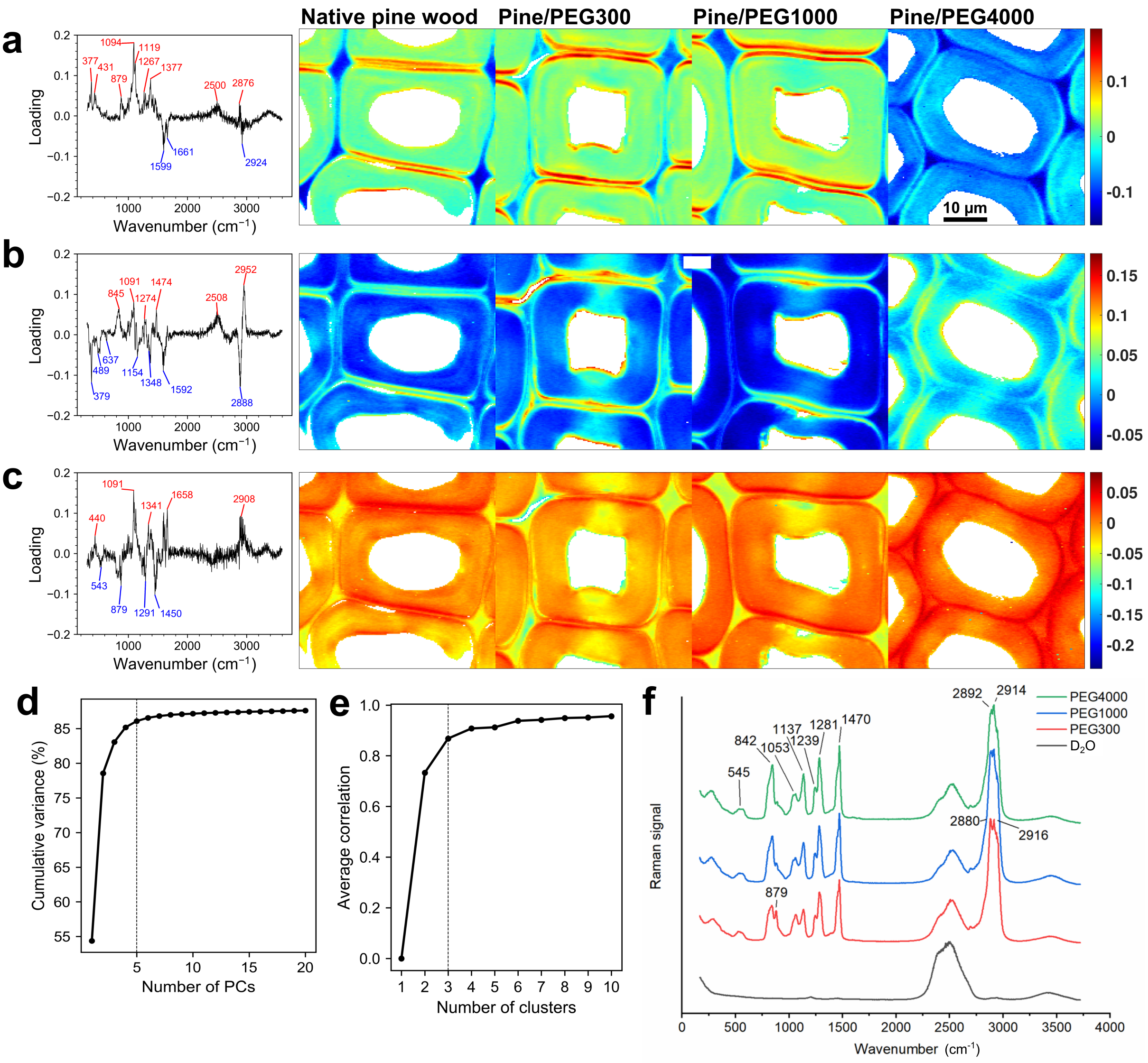

Fig. S1 Analysis of confocal Raman spectroscopy images from pine wood in D_2O with and without PEG. **a** Loading plot (*left*) and score image (*right*) of principal component 3, which differentiates between orientation-dependent carbohydrate signals (positive) and lignin (negative). **b** Loading plot and score image of principal component 4, which shows bands related to carbohydrate orientation. **c** Loading plot and score image of principal component 5, which differentiates between native wood components (positive) and residual PEG and D_2O on the lumen surfaces (negative). **d** Cumulative variance as a function of the number of principal components. **e** Average correlation as a function of number of clusters. **f** Raman spectra taken from the cell lumen regions, used to assign the Raman bands of PEG.

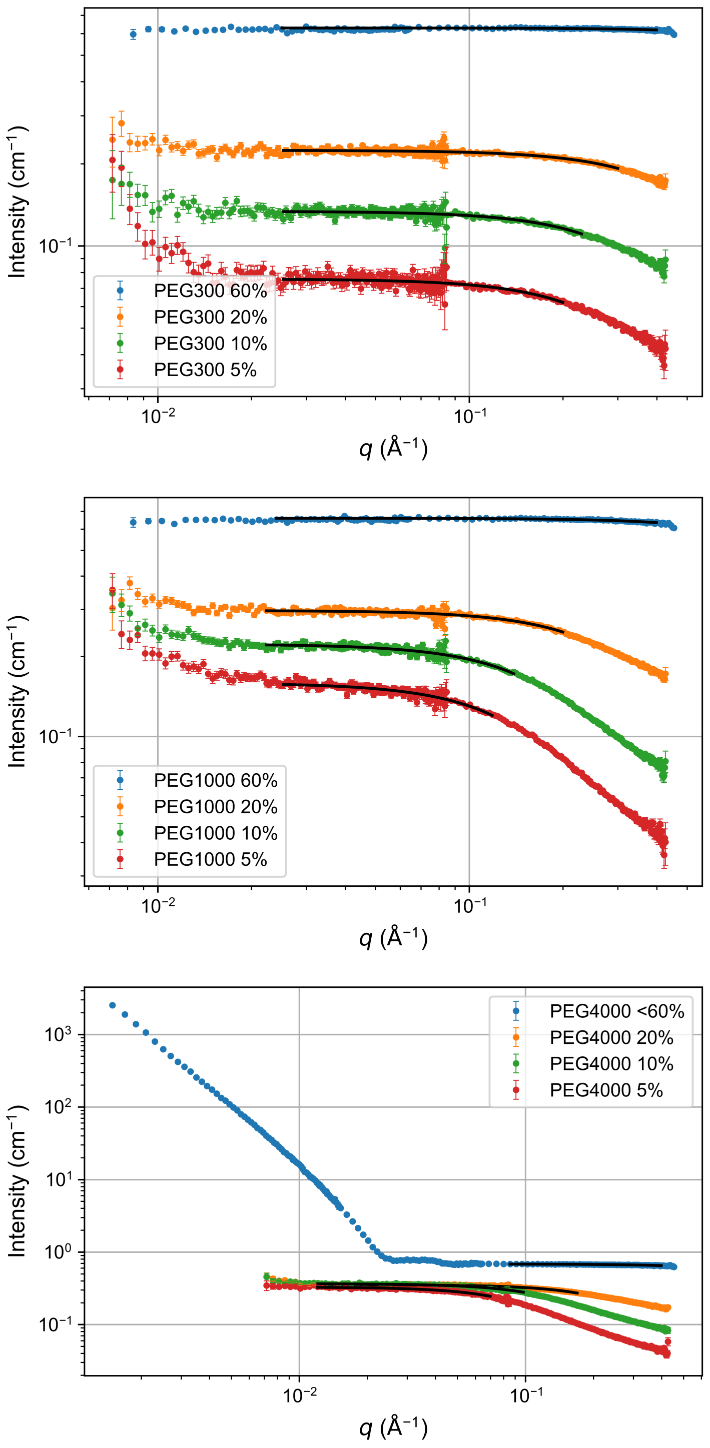

Fig. S2 SANS intensities from PEGs in D_2O, with fits of Guinier law (equation (S2), Table S2) shown with solid line.

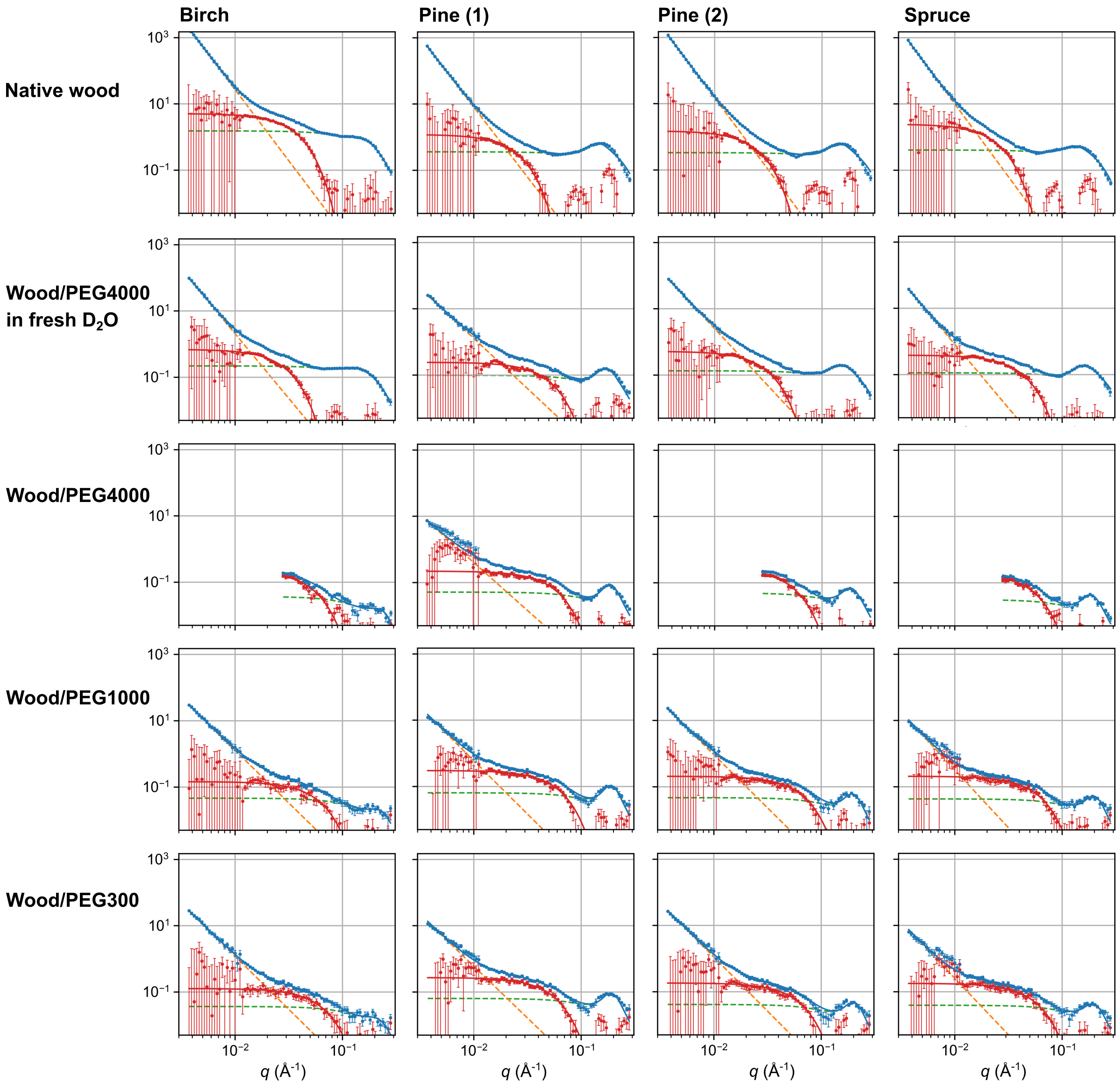

Fig. S3 Equatorial, anisotropic SANS intensities (unit cm^{-1}) and fits of equation (S1), with contributions from the different terms indicated separately as in Figs. 2b,d of the main article. The contribution assigned to the microfibril bundles (data points in *red*) was found in all samples and fitted with the Gaussian term of equation (S1).

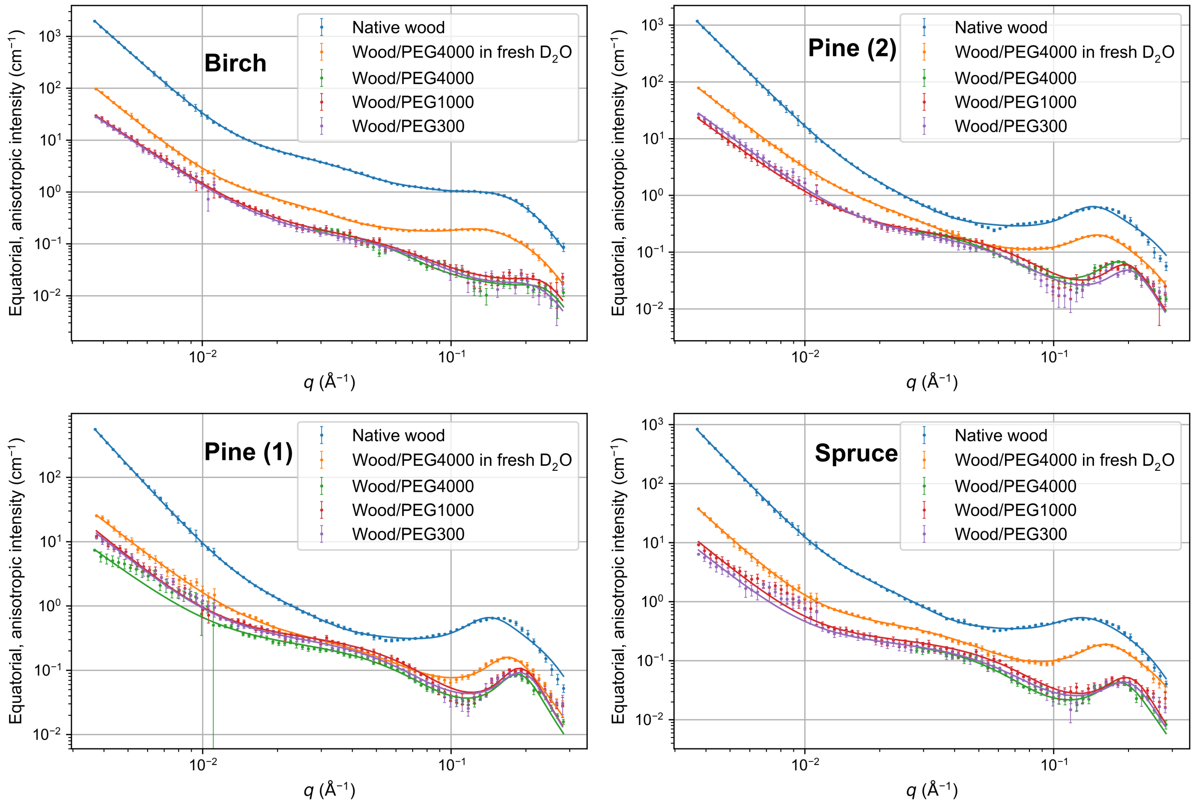

Fig. S4 Equatorial, anisotropic SANS intensities, with fits of equation (S1) shown with solid lines. All samples originating from the same wood are plotted in a single image to help visual comparison and to demonstrate the shift of the contribution assigned to the microfibril bundles (between $q = 0.01$ and 0.05 Å^{-1} in native woods) and the interfibrillar correlation peak (around $q = 0.15$ Å^{-1} in native woods) to higher q values with PEG impregnation.

References

[1] Penttilä, P. A., Rautkari, L., Österberg, M. & Schweins, R. Small-angle scattering model for efficient characterization of wood nanostructure and moisture behaviour. *J. Appl. Crystallogr.* **52**, 369–377 (2019).

[2] Hashimoto, T., Kawamura, T., Harada, M. & Tanaka, H. Small-angle scattering from hexagonally packed cylindrical particles with paracrystalline distortion. *Macromol.* **27**, 3063–3072 (1994).

[3] Jakob, H. F., Tschegg, S. E. & Fratzl, P. Hydration dependence of the wood-cell wall structure in *Picea abies*. a small-angle X-ray scattering study. *Macromol.* **29**, 8435–8440 (1996).

[4] Nishiyama, Y., Langan, P., O'Neill, H., Pingali, S. V. & Harton, S. Structural coarsening of aspen wood by hydrothermal pretreatment monitored by small- and wide-angle scattering of x-rays and neutrons on oriented specimens. *Cellulose* **21**, 1015–1024 (2014).

[5] Guinier, A. & Fournet, G. *Small-Angle Scattering of X-Rays* (John Wiley & Sons, 1955).

[6] Porod, G. General theory in *Small Angle X-ray Scattering* (eds. Glatter, O. & Kratky, O.) 17–51 (Academic Press, 1982).

[7] Plaza Rodriguez, N. Z. *Neutron scattering studies of nano-scale wood-water interactions*. Ph.D. thesis, University of Wisconsin-Madison, U.S.A. (2017).
